# Terson Syndrome in Two Infants: Case Report and Literature Review

**DOI:** 10.7759/cureus.75102

**Published:** 2024-12-04

**Authors:** Muhannad M Alobaid, Abdulmajeed I Alhaidari, Mohammad I Adhi, Mohammed Nabeel Refka

**Affiliations:** 1 Ophthalmology, Prince Sultan Military Medical City, Riyadh, SAU; 2 Ophthalmology, King Khaled Eye Specialist Hospital, Riyadh, SAU; 3 Department of Ophthalmology, King Abdulaziz Medical City, National Guard Health Affairs, Riyadh, SAU

**Keywords:** head trauma, pediatric retina, retina hemorrhage, retina surgery, terson syndrome

## Abstract

We report the presentation and outcome of Terson syndrome in four eyes of two infants in a tertiary hospital in Saudi Arabia.

This is a retrospective report of two infants with Terson syndrome due to accidental traumatic head injuries. Intraoperative screenshots of the posterior pole were taken for both cases.

A 4-month-old healthy boy had Terson syndrome following a motor vehicle accident. The patient underwent pars plana vitrectomy (PPV) for both eyes. Eventually, he had poor visual outcomes due to macular scars, sclerosed retinal vessels, and a thick internal limiting membrane (ILM). The other case was a 10-month-old boy who had Terson syndrome after recurrent falls. The patient underwent PPV for both eyes. Later, the patient’s left retina was detached which required another intervention. Both eyes had peripapillary atrophy and epiretinal fibrosis.

Terson syndrome is an uncommon condition in the pediatric population. Generally, patients with Terson syndrome have good visual outcomes. In this case report, we describe an incidence of Terson syndrome in two infants following traumatic head injuries who had poor visual outcomes after surgical intervention due to macular scars, posterior pole fibrosis, sclerosed retinal vessels, and optic disc atrophy.

## Introduction

The association between subarachnoid hemorrhage and vitreous hemorrhage was first noted in 1881 and has been known since then as Terson syndrome. Since that time, the definition of the syndrome has become more inclusive of other clinical entities. It is currently recognized as an intraocular hemorrhage in association with intracerebral hemorrhage, subarachnoid hemorrhage (SAH), or traumatic brain injury [1].

Terson syndrome was thought to be rare, as there were only 16 case reports by the year 1952, all of which had adult patients. However, since the first description of the clinical entity in 1881, the more inclusive definition of Terson syndrome has allowed more recognition and reporting of cases. Two prospective studies estimated the incidence of Terson syndrome at 16 to 27% of SAH patients [2,3]. Causes of Terson syndrome can be both traumatic and nontraumatic. Most cases of Terson syndrome have a good visual prognosis. However, in this report, we describe the occurrence of Terson syndrome in two infants that had poor visual outcomes.

## Case presentation

First case

A 4-month-old healthy male infant was referred and admitted to our hospital for further management after sustaining a motor vehicle accident (MVA) one month earlier in which he was restrained in a safety seat. After the accident, the patient developed abnormal tonic movement with up-rolling eyes and was taken to a local hospital where he was admitted to the intensive care unit and intubated.

In our hospital, brain computed tomography (CT) scan and magnetic resonance imaging (MRI) showed a left chronic subdural hematoma accompanied by a non-displaced fracture over the left parietal temporal lobe and communicating acute hydrocephalus which required ventriculoperitoneal shunt insertion 1 day after the admission.

During the ophthalmic examination, the patient did not fixate or follow with both eyes. There were mid-dilated pupils with very sluggish reactions and bilateral abnormal red reflexes. Dilated fundus examination revealed bilateral and dense organized vitreous hemorrhage obscuring the posterior pole, and peripheral intraretinal hemorrhage. Neural insult to the optic nerve could not be ruled out based on visual evoked potential test. Anterior segment examination was normal, except for bilateral small sub-capsular opacities which were most likely congenital.

**Figure 1:**
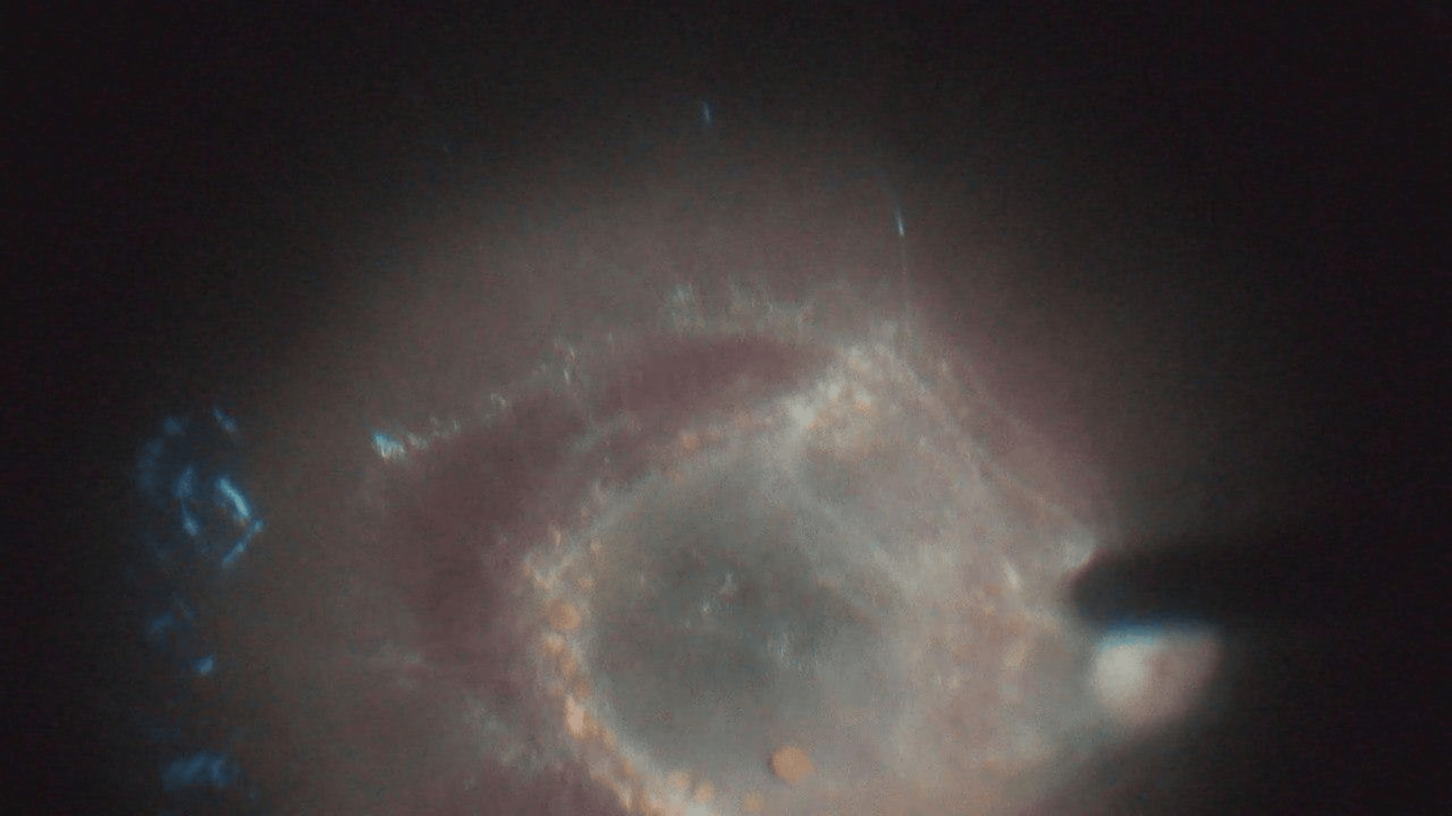
Sub-retinal hemorrhage, sub-internal limiting membrane hemorrhage, peripheral retinal hemorrhage, and hemoglobin spherules

The patient underwent pars plana vitrectomy (PPV) for the left eye which revealed, after vitreous hemorrhage removal, a big macular scar with thick crystal precipitate at the bed of the scar, sclerosed retinal vessels, and thick internal limiting membrane (ILM) resembling epi-retinal membrane (ERM). There was sub-retinal hemorrhage, sub-ILM hemorrhage, peripheral retinal hemorrhage, and hemoglobin spherules covering the temporal part of the macula (Figure 1). Specimens from a few brownish spherules from the sub-ILM membrane were obtained for histopathologic evaluation, which revealed a small number of extravasated red blood cells.

Three days later, PPV was done for the right eye which showed almost similar findings as the left eye.

Three months later (last documented visit), examination showed poor fixation and following, bilateral clear visual axis, good red reflexes, pale optic discs, and macular scars.

Second case

A 10-month-old male was a known case of left hypoplastic heart syndrome on furosemide, status post-Norwood procedure with Sano shunt at the age of 18 days. He was also on levetiracetam for seizures that started after a fall at the age of 8 months. He was brought to the emergency department after a fall from a 1-meter height. According to the mother, he rolled over, fell on his back, and hit his head. After the event, he was noted to be hypoactive, had nystagmus, and stopped following objects. There was no history of vomiting or loss of consciousness. Brain CT scan and MRI showed left frontal acute subdural hematoma with right frontotemporal subdural hematoma and generalized loss in brain volume.

On ophthalmic examination, 12 hours after the fall, the patient was not fixating or following with both eyes. Pupils were mid-dilated and non-reactive, and no red reflexes could be appreciated. Dilated fundus examination revealed bilateral organized old vitreous hemorrhage with no view of fundi that could be from previous incidence or fall. Anterior segment examination was normal. B-scan showed bilateral vitreous hemorrhage and funnel-shaped detached posterior hyaloid which was still attached to the disc and surrounding retina.

**Figure 2:**
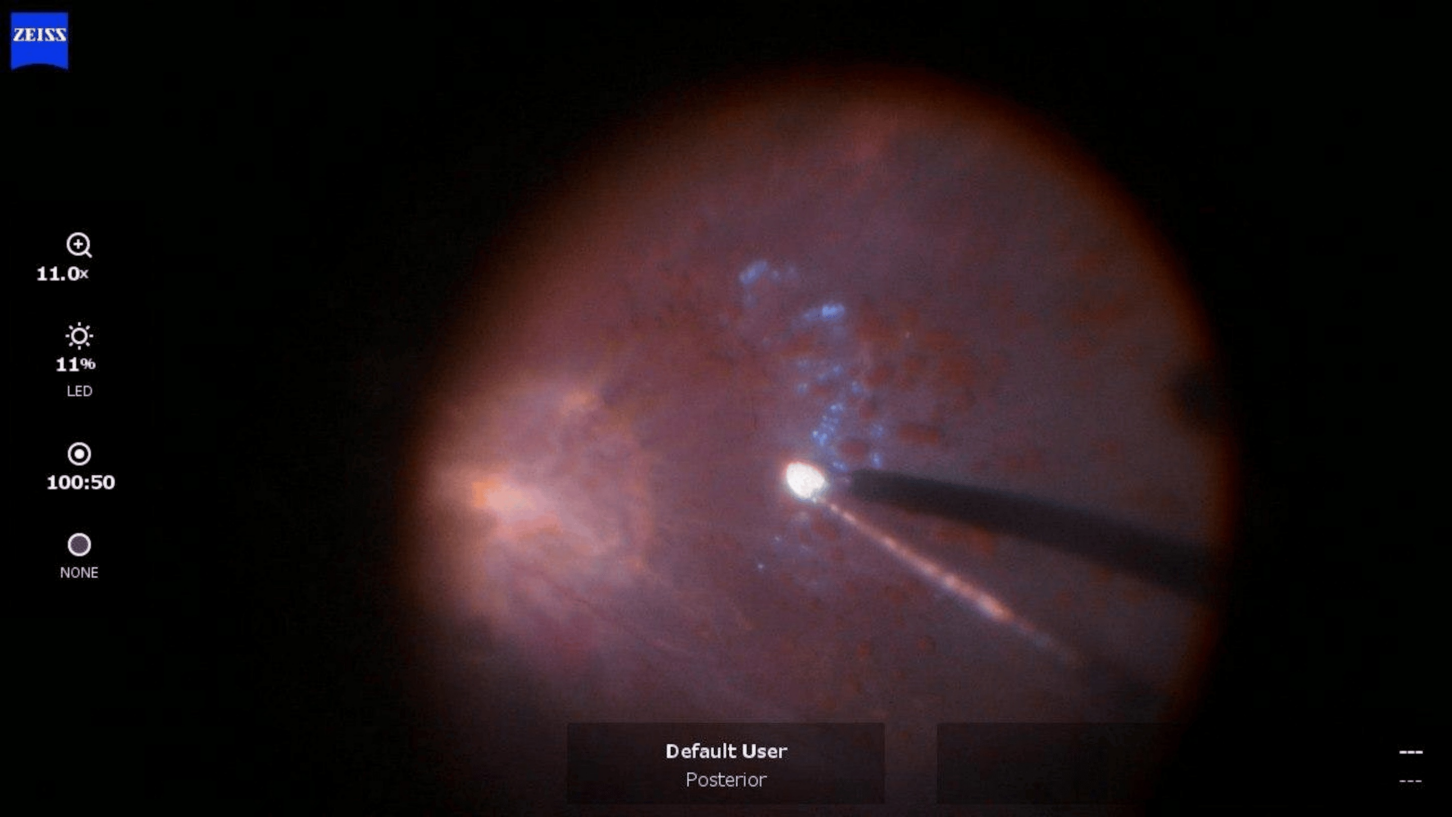
Preretinal pigmented spherules

The patient underwent PPV+ sulfur hexafluoride (SF6) for the right eye. During surgery, the area overlying and adjacent to the optic disc appeared fibrosed and there was a dense organized vitreous hemorrhage, and preretinal pigmented spherules (Figure 2). The posterior part of the vitreous was found to be densely adherent to the adjacent retina, thus, it was trimmed and detached. During the process, two iatrogenic retinal breaks complicated the procedure one disc diameter inferior to the disc which were treated with lasers after flattening the retina under air. Peripheral retinal hemorrhage was also found. The macula and peripapillary area showed areas of un-removable epiretinal membrane.

On follow-up 3 weeks later, the patient was stable, fixating, and following with his right eye with good red reflex. A dilated fundus examination showed peripapillary scars.

**Figure 3:**
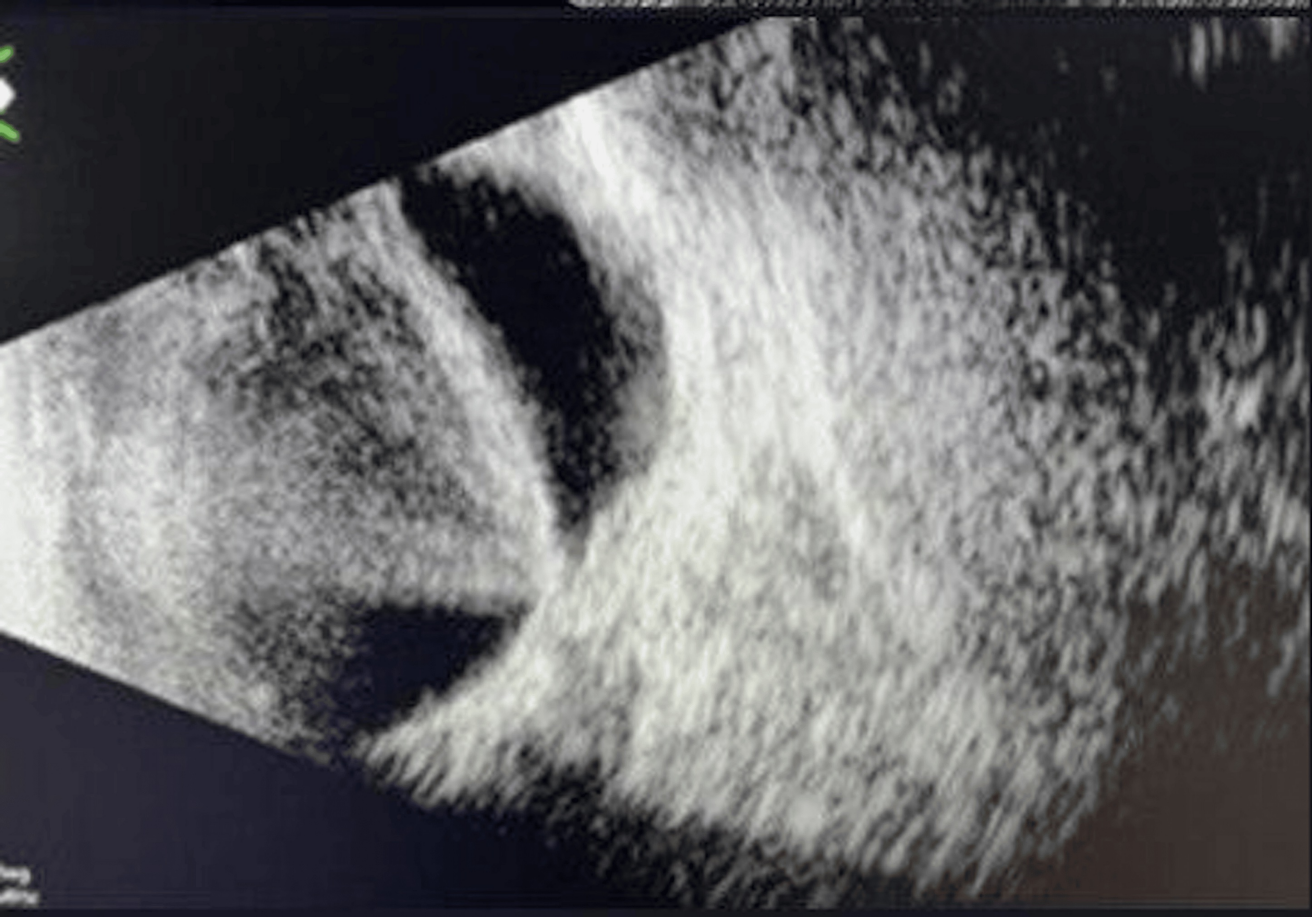
Dense vitreous opacity and posterior vitreous detachment

Two months later, the b-scan showed the right retina to be flat. The left eye showed dense vitreous opacity with thickened posterior vitreous detachment attached to the disc (Figure 3). The patient underwent left PPV+SF6 20% gas injection. There was a dense vitreous hemorrhage and a thick vitreous that was adherent to the disc. The vitreous was surgically detached and there was a shining sheath of the vitreous lying on the retina giving the impression of vitreoschisis. The peripapillary vitreous adhesions were strong and needed to be peeled and cut. Like the right eye, two fine retinal breaks in the inferior retina about one disc diameter inferior to the disc were noted. After vitreous removal, the peripapillary retina was found to be detached. The retina was flattened under air and small retinal breaks were lasered after with air-SF6 exchange was carried out.

One month after the left PPV, the b-scan showed a flat retina in the right eye and retinal detachment in the left eye. The patient underwent PPV + phacoemulsification and intraocular lens insertion with silicone oil infusion which was removed four months later. The retina was found to be flat with extensive peripapillary atrophy and epiretinal fibrosis in the posterior pole.

Both eyes underwent vitrectomy. The retinal findings and surgical scenarios were similar. Both showed dense vitreous hemorrhage with a strongly adherent vitreous to the optic nerve and surrounding retina. In both eyes, fine breaks in the inferior retinae were fixed with endolaser and SF6 gas tamponade. Epiretinal membranes on the macula were observed and these membranes were very adherent to the macula and couldn’t be removed, and pigmented spherules were seen spread on the retinal surface.

The course of the left eye was complicated by retinal detachment which was fixed by another PPV intervention and silicone oil injection.

## Discussion

Terson syndrome in the pediatric population is rare. Out of 159 publications in the literature (using PubMed Central), only 14 were on pediatrics and written in English [4-17]. The youngest case described in the literature is of a 35-day-old infant [8]. The reason behind the rare occurrence of Terson syndrome in this group of population is yet to be known. Even though some etiologies such as arteriovenous malformation (AVM) are rare in the pediatric population, a review of the literature reveals that even in relatively common entities such as subarachnoid hemorrhage and raised intracranial pressure, intraocular hemorrhages are rare in children [4]. Better autoregulation of retinal vasculature, hormonal factors, and a more restrictive communication between the optic sheath and intracranial space were three mechanisms suggested to be the reason why Terson syndrome in pediatrics is rare [5].

By 1952, 15 of the 16 adult case reports were the consequence of ruptured aneurysms and one resulted from trauma [4]. Several mechanisms have been postulated as the underlying cause of Terson syndrome. The most probable theory is that the cause of the syndrome is a rapid increase in intracranial pressure, which would result in the accumulation of cerebrospinal fluid around the optic nerve sheath. This accumulation will result in the compression of the optic nerve sheath on the central retinal vein, which would in turn lead to retinal venous hypertension and vein rupture [6].

In literature, among the 14 pediatric reports, causes of Terson syndrome were perinatal ischemic stroke [5], ruptured aneurysm [6-8], traumatic head injury [4,9,10], birth trauma [11], nontraumatic intracranial bleedings related to leukemia and intracranial tumor removal [12,13], dengue hemorrhagic fever causing viral retinitis and thrombocytopenia [14], cortical venous sinus thrombosis [15], venous malformation [16], and shaken baby syndrome [17]. In our report, both of our cases were secondary to accidental traumatic head injuries.

Fifty percent of vitreous hemorrhages do not resolve spontaneously after 19 months [18]. Adults with dense vitreous hemorrhage or bilateral involvement with poor vision are candidates for vitrectomy [19]. In pediatrics, earlier intervention is advised to prevent the possibility of developing amblyopia [19]. Generally, several factors dictate the optimal time of operation, such as bilateral involvement or the patient’s age [19]. In a review of the literature, conservative management was enough in approximately 50% of the pediatric cases, whereas in our report, both cases required PPV.

The outcome of Terson syndrome is mostly good, whether it’s managed conservatively or surgically. In one study of 11 cases of Terson syndrome due to subarachnoid hemorrhage, spontaneous regression of vitreous hemorrhage was seen in 4 patients, and 7 eyes of 6 patients reported median improvement from hand motion vision to 20/25 following pars plana vitrectomy [1]. In another study of 15 eyes, 93% had a visual acuity of 20/40 after undergoing PPV for vitreous hemorrhage [20].

In our report, both cases had poor outcomes. In the first case, even though visual axes were clear for both eyes with good red reflexes, there were a big macular scar and sclerosed retinal vessels in the left eye. The right eye had a small macular scar, sclerosed retinal vessels, and a pale disc. Those abnormalities contributed to the poor fixation and following in this case. In the second case, there was posterior pole fibrosis which contributed to the poor prognosis.

## Conclusions

Terson syndrome is an uncommon condition in the pediatric population. Generally, patients with Terson syndrome have good visual outcomes. In this case report, we describe an incidence of Terson syndrome in two infants following accidental traumatic head injuries who had poor visual outcomes after surgical intervention due to macular scars, posterior pole fibrosis, sclerosed retinal vessels, peripapillary atrophy and, possibly, optic disc atrophy.
